# Assessing the publication output in the field of forensic science and legal medicine using Web of Science database from 2011 to 2020

**DOI:** 10.1080/20961790.2021.2002525

**Published:** 2022-07-22

**Authors:** Bedirhan Sezer Öner, Metin Orbay

**Affiliations:** a Department of Forensic Medicine, Faculty of Medicine, Amasya University, Amasya, Turkey; b Department of Mathematics and Science, Faculty of Education, Amasya University, Amasya, Turkey

**Keywords:** Bibliometrics, citation analysis, forensic science, legal medicine, Web of Science

## Abstract

The aim of this study was threefold. First, it analyzed the characteristics of the publication outputs for the Legal Medicine (LM) category using the Web of Science (WoS) database during 2011–2020. Second, it discussed the distribution of the papers for the 25 most productive countries/regions in terms of quality and quantity, such as the *h*-index and GDP *per capita*. Finally, it investigated the trend and temporal stability of the journal impact factor (JIF) and determined the percentage of the journal self-citations. The findings suggested that the number of papers, the average number of pages of the papers, the average number of cited references in the papers, the average number of authors per paper, the percentage of open access papers, as well as international and domestic collaboration tended to increase regularly. However, the productivity was limited when compared to the whole WoS database, since there was no significant change in the number of the journals. The countries/regions with the highest number of publications were not those that made the most impact in terms of the widespread impact of the publications. The level of international cooperation and the funding for the research had dramatic impact on the visibility of papers. The average JIF has increased significantly while the journal self-citation rates have decreased in a similar way. The journals have had very stable (have not fluctuated) impact factors over time. During the period studied, the journals with the higher impact factors (Q1, Q2) published many more papers than journals with the lower impact factors (Q3, Q4).

## Introduction

The search for solutions by analyzing problems with interdisciplinary cooperation and collaborative culture emerged in the 20th century and has turned into an accepted model today. In this context, medicine is the branch that best maintains joint cooperation in all of its fields, especially Forensic Science and Legal Medicine (LM), which use medical knowledge and methodology for the resolution of legal questions and problems experienced by individuals and society [1]. Throughout this study, LM was chosen as the umbrella concept that will from now on to refer to both “Forensic Science” and “Legal Medicine” [2]. It has particularly reached a certain standard both in scientific studies and in the field of application and has come to an important point together with interdisciplinary and multidisciplinary scientific approaches in conjunction with the rapid developments in the fields of science and technology [1]. Jones [3] pointed out that 11 sections of the American Academy of Forensic Sciences in the USA, which is the leading country in the LM field, currently has more than 6 500 members. On its way to success, academic journals play a primary role among the official communication languages of science in the process of building, disseminating, and using knowledge [4,5]. As a result of the increasing interaction with other disciplines, wide content and internationalization, this holds true for the field of LM as well. Thus, as in the other sciences, it would be hard to imagine the field of LM without academic journals [6]. Meanwhile, the discipline is relatively small when compared with other medical fields in terms of academic publishing although the first journals published articles about LM, crimes, and criminology in Germany towards the end of the 19th century [7].

The number of researchers and journals has been increasing over time and this competitive environment has led to some novel discussions of “publish or perish!” and “quality or quantity” [8]. Therefore, following the publications and analyzing the widespread effects of them in academic journals in the LM field is a prerequisite to understanding the level of expansion of the field and the cooperation with other disciplines. In this sense, analyzing the citation relationship networks between the publications based on various criteria would allow this need to be met. In this respect, every effort is made to understand the present and make inferences between the past and the future, and this gains importance day by day.

Developments in information technologies have made it possible to access information easily and cheaply, and the amount of accessible information has increased exponentially with each day passing [9]. However, it is very important to obtain the desired information with source security and up-to-dateness by ensuring that it does not remain as a data pile without any benefit. For this reason, classifying the data rather than working with the data in bulk, provides the opportunity to analyze them better and reach the correct, reliable, and sufficient information needed.

One of the methods that can be used for this purpose is the bibliometric analysis method, which was first defined by Pritchard [10]. Bibliometric studies are those that reveal the current status, orientation, and development of studies in the current literature related to a discipline [11]. These studies include mathematical or statistical analyses of the literature according to the distribution of citations, people, topics, countries, or publication types such as books and articles. It provides an opportunity to have a deep understanding of a research field [12]. It also makes it possible to reveal the interest in a science, the tendency towards a variety of topics in that discipline, the change in these trends, and the most cited topics, authors, and publications [13].

Nowadays, the articles published in journals in the Web of Science (WoS: Web of Science Core Collection by Clarivate Analytics) database are predominantly accepted in the academic community as quality research, and as a result, this database is frequently used in bibliometric analysis [14–17]. Liu [18] showed that there was a boom in the number of papers using the WoS database between 2009 and 2018. By sorting out the studies by category, they showed that four of these top five categories were medicine/health-related, which reflected the wide application of WoS in medicine/health research. Apart from being an abstract database, WoS is a citation database, which calculates the citations received by every single paper indexed in the database. From these citations, several citation indices have been created, the most popular of which is the journal impact factor (JIF). The JIF is defined as the number of citations in the current year to items published in the previous 2 years, divided by the total number of scholarly citable items published in those same 2 years [19]. Although there is great interest in the JIF within the research ecosystem, the inclusion of the journal self-citation, skewness in citation distribution, and the citation window being limited to 2 years, make the use of the JIF an intensely discussed topic [20]. As a result of this discussion, the 5-year JIF and JIF without self-citations have been calculated and are now available in the Journal Citation Report (JCR).

New indicators have been developed to be used as an alternative or in combination with the JIF [20]. Among these alternative indicators, perhaps the *h*-index is the most popular [21], which was originally developed for evaluating researchers and attracted great interest in the literature in a very short time [22]. According to the definition by Hirsch [21, p. 16569], “*A scientist has index h if h of his or her N_p_ papers have at least h citations each and the other (N_p_–h) papers have ≤ h citations each*”. After a short time, the *h*-index has been extended to measure the academic productivity and impact of journals and of universities and other research institutions. Many academics have used the *h*-index for ranking researchers and research groups in a specific field or country [23–27].

In the WoS database, journals in the LM field are indexed in the “Medicine, Legal” category (from now on the LM category) in the Science Citation Index Expanded (SCIE). The LM category is described as “*covering resources on all aspects of medical legal issues, including government regulations and policies, malpractice, toxicological and pharmacological regulations, clinical therapeutic patents and other critical legal issues at the interface of law, medicine, and healthcare. The category also covers resources dealing with the various branches of forensic science*” [28].

## Recent studies using bibliometrics methods in the LM field

The first bibliometric study in the LM field focussed on the evaluating the work of researchers by citation analysis and was first published with the title “*The hottest in forensics: citations tell whodunnit*” in the Science Watch report [29]. In this report, the most productive institutes, the most cited papers, and some bibliometric indicators about the authors were given by taking into account the leading nine international journals of the field between 1981 and 1993. This report was the first quantitative study to evaluate journals in the LM field. As the LM field had characteristics different from other disciplines, it was criticized for certain issues such as the low impact value of journals and the high rate of self-citations or negative citations [30,31].

Recently, a lot of analyses have been carried out using bibliometric methods for the LM and related sub-fields. In this section, it is essential to underline some of the studies conducted in the last two decades have appeared in the literature. Jones [32] reviewed the JIF for the LM field and the toxicology journals and concluded that the impact factors of these journals were low because the visibility and size of the circulation of these journals were limited. Jones [33] conducted research into the most active researchers who contributed to the development of the LM field, the most cited papers, and the journals which published these papers. Jones [6] found that the relatively low JIF in the LM field was due to the small size of the field, fewer active researchers, and less pressure on them to publish more. Sauvageau et al. [34] focussed on the evolution of two North American journals in the LM field and found that the number of papers per year and the average number of authors per paper over the 25-year interval both increased almost two-fold. Jayapriya and Sivaraman [35] pointed out that despite the increase in the number of publications in the LM field, the trend was slow, and emphasized that the USA was the leading country. Ravichandra Rao et al. [36] indicated that while both India and China had a significant presence in the global literature in the LM field, the quality of research (in terms of citations received) was greatly below that of the global average when measured in terms of widely used indicators.

Tran et al. [37] conducted a bibliometric study that focussed on the field of child abuse, which is directly related to the LM field. Lei et al. [38] looked at the global productivity in the field of forensic anthropology, the most productive researchers, the most cited studies, and the keyword analyses used extensively by the researchers. Shi et al. [39] focussed on medical malpractice in the field LM and searched for the most productive countries, journals, authors, and journals with the highest impact. Lei et al. [40] focussed on studies in the field of forensic entomology and presented arguments on the variations of papers per year, contributions of the countries to the field, prominent countries, journals, most cited studies, and keywords. Demir et al. [41] investigated the most active journals, popular fields of study, and the relationship between countries’ Gross Domestic Product (GDP) and scientific productivity in the LM field. Jones [3] re-focussed on the most cited scientists in the LM field and analyzed these scientists in detail according to their fields of study, *h*-index, self-citation rates, and whether or not the researcher was the first author.

## Purpose of the present study

The current study aimed to answer the questions below by conducting a bibliometric analysis of the papers published in the LM category between 2011 and 2020. WoS database was used as the data source. The research questions (RQ) were as follows:

**RQ1:** What is the number of papers, the average number of pages of the papers, the average number of cited references in the papers, and the average number of citations and authors per paper and what is the percentage of open access papers as well as international and domestic collaboration in the papers?

**RQ2:** Which are the most productive countries? What is the relationship of the *h*-index with the average citation values of the publications and the GDP *per capita* (US$) of these countries, and the number of papers? What is the level of national and international cooperation among the total papers of these countries? What is the level of funding in the studies?

**RQ3:** What is the trend and temporal stability of the JIF values in the LM category? What are the self-citation rates of the journals?

## Methodology

The data were collected from the LM category of the SCIE in the WoS Core Collection on July 5th, 2021. The timespan was from 2011 to 2020. The document numbers, publication years, paper types, countries/regions, funding agencies, languages, sources, and authors were collected *via the Results Analysis* application. The citing papers were obtained from *the Citing Report*. The information on the papers required was also imported into Microsoft Excel 2016 (Microsoft Corporation, Redmond, WA, USA) for data processing and SPSS Version 25 (IBM Corporation, Armonk, NY, USA) was used to calculate Intraclass Correlation Coefficient (ICC) and Pearson correlation coefficients (*r*) to find out relationship among *h*-index and some indicators with VOSviewer 1.6.13 [42].

A total of 23 957 documents were found in the first search, but after excluding unrelated documents, such as out of category and no author/anonymous documents, a total of 23 326 documents were analyzed in the LM category. Of the remaining documents, there was a total of 19 114 articles (18 143; 77.78%) and reviews (971; 4.16%), representing 81.94% of all the documents. The types of other important documents were editorial material (1 230; 5.27%), letters (1 087; 4.66%) and book reviews (859; 3.68%). Only “articles” and “reviews” document types were taken into account, which were categorized under the term “papers” throughout the study. Moreover, 2 460 of these papers were published as open access (OA), which means free to read online, either on the publisher website or in an OA repository [43]. When the language of the papers was analyzed, two languages appeared, respectively, English (18 592; 97.27%) and German (522; 2.73%). The number of journals published in the LM category varied between 15 and 17, and a total of 13 journals were regularly indexed during the period 2011–2020.

## Results and discussion

In this section, the findings of the research questions were presented. The interpretations of these findings were discussed in comparison to the related literature.

### Results and discussion for RQ1

Table 1 presents the number of papers (PN), the average page number (APN) in papers, the average cited references (ACR), the average number of authors per paper (APP), the percentage of open access (OA%) papers as well as international and domestic collaboration (IC%, DC%). The values given in the brackets were those obtained when only the articles were taken into account.

**Table 1.** Characteristics of the paper outputs for the legal medicine (LM) category from 2011 to 2020.

Year	JN	PN	APN (A)	ACR (A)	APP (A)	OA%	IC%	DC%
2011	15	1 601	6.74 (6.70)	27.74 (26.72)	4.19 (4.21)	4.99	16.78	35.52
2012	16	1 763	7.02 (6.92)	28.65 (27.50)	4.38 (4.43)	6.13	16.90	35.03
2013	16	1 848	6.99 (6.91)	29.53 (28.23)	4.43 (4.52)	6.98	16.51	38.06
2014	15	1 805	7.39 (7.36)	30.73 (29.61)	4.78 (4.82)	10.64	20.53	37.05
2015	15	1 958	7.50 (7.47)	30.93 (29.72)	4.75 (4.79)	9.81	20.15	39.07
2016	15	2 032	7.61 (7.57)	30.82 (29.88)	4.95 (4.99)	14.76	20.23	39.58
2017	16	1 915	7.94 (7.88)	32.93 (31.61)	4.78 (4.80)	14.57	21.66	39.74
2018	17	2 077	8.25 (8.21)	35.49 (32.99)	4.93 (4.98)	17.04	19.74	40.53
2019	16	2 056	8.08 (7.92)	33.73 (31.21)	5.03 (5.12)	17.05	18.12	43.62
2020	17	2 059	8.57 (8.52)	35.58 (33.89)	5.15 (5.16)	23.12	20.44	41.48

JN: journal number; PN: paper number; APN: average page number; ACR: average cited reference; A: article; APP: author per paper, OA: open access, IC: international collaboration; DC: domestic collaboration.

**Figure 1.**
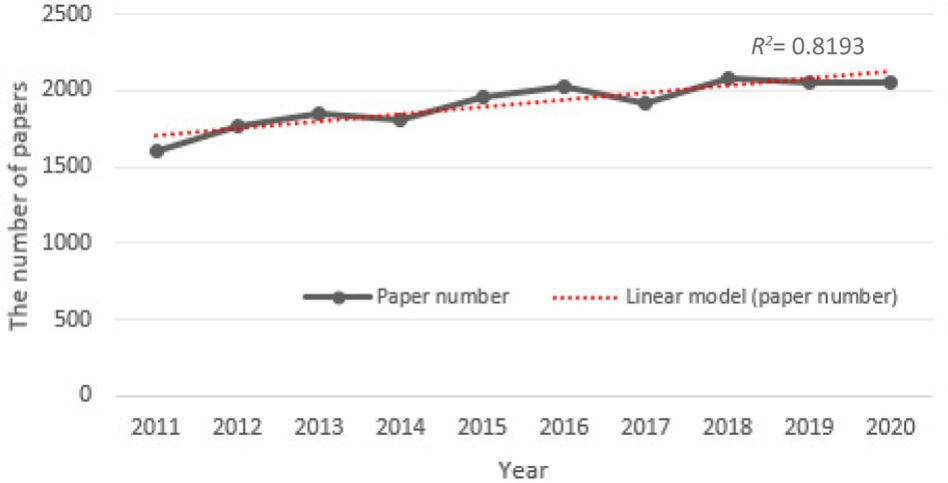
The number of papers for the legal medicine (LM) category during 2011–2020.

There was no significant change in the number of journals indexed in the LM category. Despite this, the number of published papers tended to increase regularly. The change in the total number of papers in the above-mentioned period is shown in Figure 1. A significant correlation was found between the time period and the number of papers published (*R^2^= 0.819; r = 0.905, P = 0.0003*). The growth pattern was represented by a linear model.

It is not surprising that the number of papers in the LM category has increased this way. Likewise, Hu et al. [9] found that the whole WoS Core Collection showed a yearly increase. In fact, an exponential growth curve provided a better fit, even though the increases were roughly linear (*over R^2^ = 0.95*). It showed that productivity was limited in the LM category when compared to the whole WoS database. This can be attributed to the small size of the field, fewer active researchers, and less pressure on the researchers to publish more, as Jones [6] stated. What should be emphasized here is that the number of journals indexed in the WoS database in the LM field has remained stable. Although the time period is quite large, the limited number of journals can be interpreted as the WoS database being very conservative in this regard. On the other hand, in 2015, a decision was made to expand the WoS database by taking into account more geographically and socioeconomically diverse countries or developing disciplines, and a new index, called the Emerging Sources Citation Index (ESCI), was created [44]. There are six ESCI journals (from China, Canada, Egypt, Iran, France, and Poland) available in the LM category. These journals do not have impact factors. However, the JCR citation counts include ESCI citations, and consequently contribute to other JIFs. Moreover, in the continuously growing, dynamic and diverse literature, the ESCI provides WoS users with extended possibilities to explore emerging research areas [45]. It should not be forgotten that the papers published in these journals attract a wide readership by appearing in the WoS [46]. Moreover, since the LM field is a multidisciplinary field, which covers many branches of pure, applied, and biomedical sciences, researchers submit the results of their research for publication to a wide range of academic journals, not only to LM journals [3].

As can be clearly seen from Table 1, the APN of the published papers and the ACR steadily increased from 6.74 to 8.57 and from 27.74 to 35.58, respectively. On the other hand, the average number of APP showed that almost one more author was added as a co-author for the period.

As stated in the Methodology section, it should be remembered that articles and reviews were gathered under one roof as papers within the scope of this research. The main reason for giving this reminder here is that since the number of pages of the reviews, the number of references they cited and the average number of citations they received were mostly higher than the articles [47], the results obtained only for the articles when the calculation was repeated are given in the brackets in Table 1, excluding the reviews. When the data in Table 1 are re-examined for these new conditions, it is seen that the averages have decreased a little over the years, but the interpretations that were made about the relevant variables in the change trends have not changed. Since the number of pages, the number of cited references, and the number of authors in the papers in the LM category tended to increase over time, this can be interpreted as the first signs that the number of citations that the paper receive will increase [48].

There is a trend towards multi-authorship in scientific communication [49–51]. Considering the number of authors per publication, the fastest change is in the field of medicine. It is known that there has been a significant increase in the average number of APP in medical journals in studies conducted by different researchers at different times [52]. The abundance of clinical research and the increasing necessity of cooperation between sub-fields are among the main reasons for multi-authorship in the field of medicine [53]. Therefore, as can be seen in Table 1, the fact that an average of one author was added to the average number of authors in papers in the LM field during 2011–2020 can be explained by the unique characteristics of the field.

There is a very close relationship between the LM field and other disciplines [3]. In fact, as can be seen in Table 2, seven of the 17 journals in the LM category are in the LM category (41.18% of journals) only, while six journals are in two categories (35.29%), two journals are in three categories (11.77%), and two journals in four categories at the same time (11.77%). The LM category is related to 10 different categories, especially Pathology and Law. Based on the WoS database studied, the first five fields in which the LM studies interact and the percentage of interaction were found as 12.03% for Pathology, 9.89% for Pharmacology & Pharmacy, 9.89% for Toxicology, 8.19% for Law, and 6.83% for Genetics & Heredity, respectively. On the other hand, the interaction frequency of journals in the LM category with other categories is given in Table 3.

**Table 2.** Impact factors (IF) and the self-citation rates (SC%) of the Legal Medicine category of journals in Web of Science (WoS) from 2011–2020.

Journal title [active quartile^a^]	WoS-C^b^	2020 IF, SC%	2019 IF, SC%	2018 IF, SC%	2017 IF, SC%	2016 IF, SC%	2015 IF, SC%	2014 IF, SC%	2013 IF, SC%	2012 IF, SC%	2011 IF, SC%	A-SC%
Forensic Sci Int-Gen [Q1]	1, 3	4.882, 33.39	–	4.884, 48.53	5.637, 47.95	3.911, 49.99	4.988, 61.25	4.604, 52.26	3.202, 40.69	3.861, 49.75	3.082, 40.40	47.13
J Law Biosci [Q1]	1, 6, 8, 9	3.583, 0	2.275, 3.47	2.431, 11.27	–	–	–	–	–	–	–	4.91
Regul Toxicol Pharm [Q1]	1, 7, 10	3.271, 7.64	2.652, 9.46	2.996, 15.92	2.815, 16.48	2.221, 17.11	2.227, 22.41	2.031, 16.10	2.142, 15.55	2.132, 14.68	2.427, 15.16	15.05
Int J Legal Med [Q1]	1	2.686, 21.93	2.222, 19.44	2.094, 20.49	2.316, 19.99	2.382, 23.43	2.862, 23.79	2.714, 25.20	2.597, 32.50	2.686, 35.63	2.587, 35.06	25.75
Forensic Sci Int [Q2]	1	2.395, 10.56	2.108, 20.92	1.990, 19.25	1.974, 17.02	1.989, 20.97	1.950, 18.31	2.140, 18.79	2.115, 18.06	2.307, 23.62	2.301, 17.95	18.54
Sci Justice [Q2]	1, 2	2.124, 14.97	2.075, 17.25	1.675, 15.82	1.845, 21.03	1.992, 13.91	1.959, 12.15	1.417, 20.61	1.415, 27.14	1.151, 47.52	1.597, 15.15	20.56
Forensic Sci Med Pat [Q2]	1, 2	2.007, 11.66	1.611, 13.28	1.815, 10.19	2.027, 14.11	1.842, 10.21	1.896, 22.05	1.983, 36.76	1.957, 29.89	2.438, 46.14	1.444, 13.43	20.77
J Forensic Sci [Q2]	1	1.832, 15.39	1.441, 13.12	1.438, 11.96	1.184, 11.23	1.127, 9.76	1.322, 12.63	1.160, 9.05	1.306, 12.48	1.244, 10.93	1.229, 17.82	12.44
J Law Med Ethics [Q3]	1, 6, 8, 9	1.718, 8.67	1.085, 8.29	0.734, 7.36	0.986, 4.97	1.223, 5.40	1.613, 9.86	1.097, 5.56	0.939, 8.41	1.169, 5.90	1.215, 3.62	6.80
J Forensic Leg Med [Q3]	1	1.614, 5.76	1.302, 8.45	1.199, 9.67	1.103, 13.42	1.135, 15.33	0.870, 17.24	0.760, 20.39	0.989, 32.76	0.856, 16.71	1.098, 26.68	16.64
Legal Med [Q3]	1, 4, 5	1.376, 8.43	1.195, 8.37	1.404, 11.68	1.254, 10.93	1.276, 22.02	1.442, 16.50	1.238, 13.65	1.441, 12.01	1.080, 16.85	–	13.38
Med Law Rev [Q3]	1, 6	1.267, 21.07	1.460, 10.96	1.577, 26.82	1.103, 17.14	–	–	–	–	–	–	19.00
Med Sci Law [Q4]	1, 6	1.266, 19.75	0.676, 6.51	0.532, 14.29	0.589, 9.51	0.689, 5.81	0.569, 12.13	0.531, 6.97	0.758, 21.77	0.484, 17.77	0.446, 13.45	12.79
Aust J Forensic Sci [Q4]	1	1.083, 12.83	1.188, 15.07	1.522, 25.56	0.941, 10.41	0.778, 12.85	0.833, 12.85	0.583, 26.07	0.704, 28.98	0.528, 36.93	0.308, 37.66	21.92
Am J Foren Med Path [Q4]	1, 2	0.921, 12.81	0.785, 23.95	0.539, 15.77	0.643, 9.80	0.648, 4.32	0.795, 7.55	0.701, 8.13	0.624, 6.41	0.883, 10.08	0.883, 1.25	10.01
Rechtsmedizin [Q4]	1	0.517, 45.84	0.592, 41.89	0.603, 52.57	0.642, 34.11	0.530, 36.04	0.324, 40.12	0.352, 70.17	0.632, 69.46	0.750, 48.93	0.814, 70.64	50.98
Rom J Leg Med [Q4]	1	0.363, 20.94	0.488, 19.47	0.480, 37.50	0.320, 43.75	0.108, 8.33	0.144, 12.50	0.233, 29.61	0.152, 23.68	0.208, 28.37	0.398, 62.06	28.62

^a^ Active quarterly is based on 2020 Journal Citation Report.

^b^ WoS-C (Category): 1: Medicine, Legal; 2: Pathology; 3: Genetics & Heredity; 4: Social Sciences; 5: Biomedical; 6: Law; 7: Toxicology; 8: Ethics; 9: Medical Ethics; 10: Pharmacology & Pharmacy.

–: NA; A-SC%: average self-citation rates (%).

The main factors resulting in the increase in the number of cited references can be explained by the expansion of the field, the increase in international cooperation, the advances in information and communication technologies, intensive work with other fields, and the increase in the number of OA publications day by day [54,55].

The most salient improvement was seen in the percentage of OA papers over time, which increased from 4.99% to 23.12% (Table 1). Especially in 2020, almost one fourth of the papers published in the LM category reached their readers as OA. As a whole, the percentage of papers published as OA in all fields has increased over the years, which can be said to be encouraging. Meanwhile, it is worth emphasizing that this trend has been observed in virtually all scientific areas in recent years since the recent EU policies, known as Plan S, require researchers who have been funded with EU grants and some external institutions to publish articles in OA journals to make the articles more publicly accessible [56,57].

On the other hand, OA papers naturally have more reads and, as a result, get more citations [43]. Such a trend can be interpreted that it would mainly solve the problem of the subscription fee, which is one of the most important problems in academic ecosystem, and it would increase the speed of dissemination of academic knowledge. This provides a big important opportunity for researchers who have limited access to academic papers. It can be expected that almost all of the journals in the LM field, as in other disciplines, will publish papers electronically in the near future [7,58]. One of the reasons for the regular increase in the number of pages of papers in the LM category may be that as the number of OA journals and papers increased over time, the cost pressure on the publishers due to the number of pages decreased, and as a result, the papers might have been more detailed and therefore the number of pages increased. In this study, the average page number of the OA papers was calculated as 10.40 (min: 8.79 (2011); max: 10.59 (2017)).

### Results and discussion for RQ2

**Figure 2.**
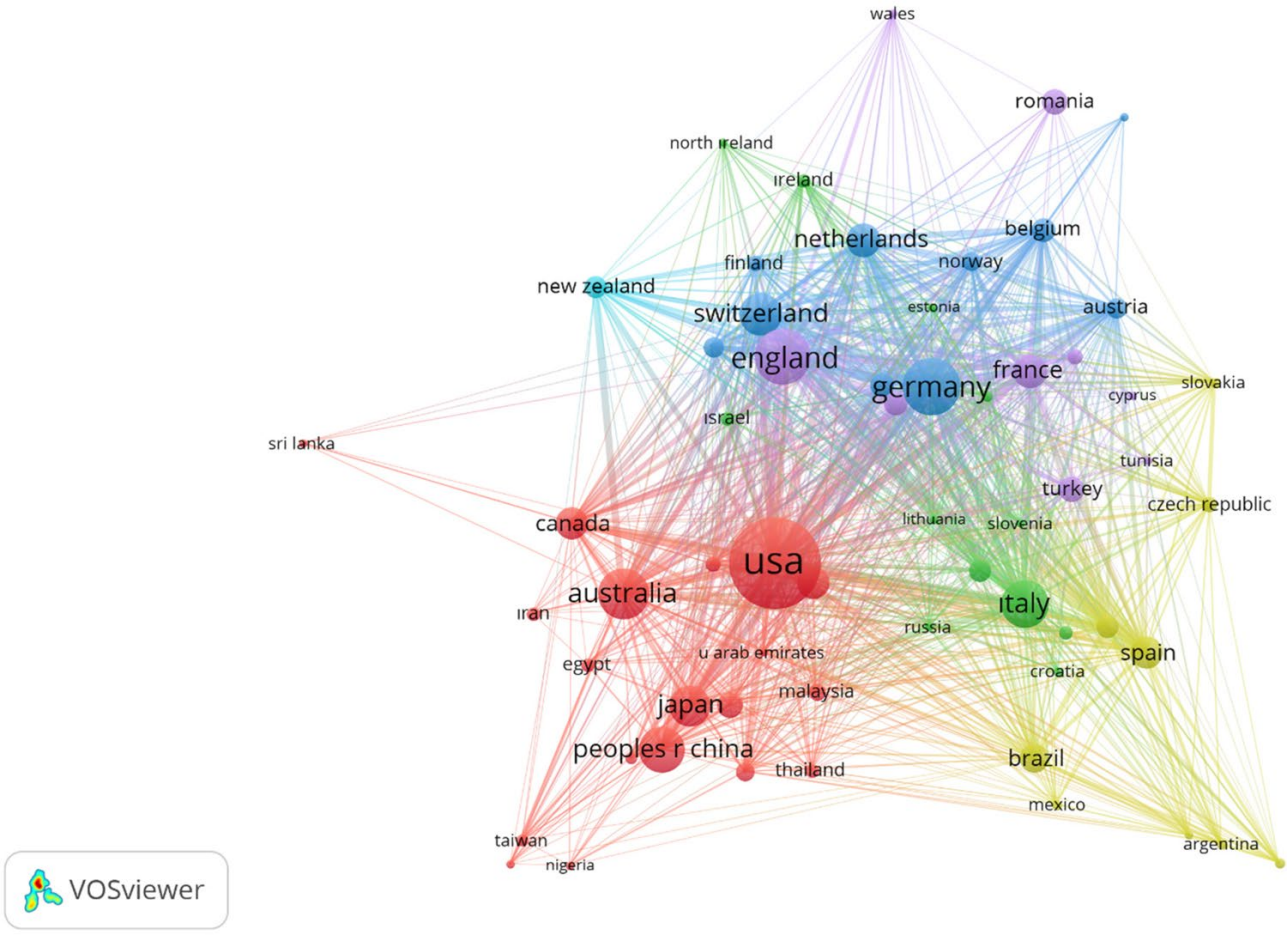
Network visualization map for collaboration between countries/regions in the legal medicine (LM) category.

Research collaboration among different countries/regions is a helpful metric for determining the wideness and impact of research. Thus, when the general situation of the papers published in the period of 2011–2020 was looked at, there was a total of 140 countries/regions that produced publications in the LM category. Figure 2 displays the network visualization map obtained from 62 countries/regions that had at least 20 publications. The size of circle in Figure 2 shows the number of papers, the thickness of the lines indicates the strength of the collaboration, and the colours indicate the cluster of collaboration. Figure 2 shows the most collaborative countries from three distinct patches: the USA, Germany, and England. In terms of international collaboration, the findings of the research revealed that the geographical location was the most essential element. The support for this assumption came from the fact that England, France, Scotland, and Wales were all in the same cluster, as were Argentina, Brazil, Chile, and Mexico, as well as Austria, Belgium, Denmark, and Finland.

**Figure 3.**
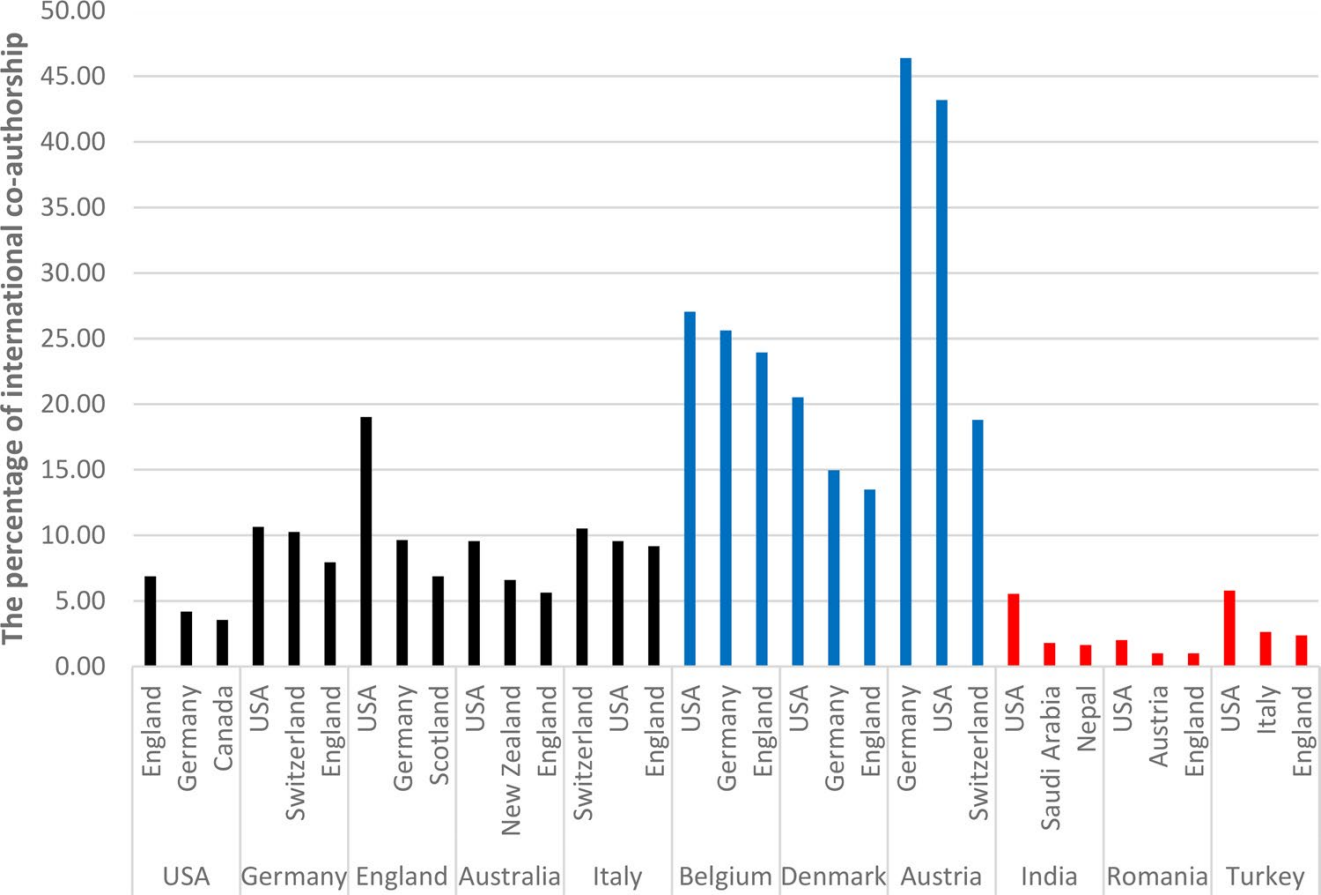
The percentage of international co-authorship of total publications for the top five countries/regions and six other countries.

Table 4 shows the distribution of the number of papers for the 25 most productive countries/regions in terms of the average number of citations per paper, the *h*-index, and the GDP *per capita* (US$) in the LM category in the 2011–2020 period.

**Table 3:** Interaction frequency (%) with legal medicine (LM) category of 17 journals in Web of Science (WoS).

Category	Interaction frequency with LM (%)^*^
Law	4 (23.53)
Pathology	3 (17.65)
Ethics	2 (11.76)
Medical Ethics	2 (11.76)
Genetics & Heredity	1 (5.88)
Social Sciences	1 (5.88)
Biomedical	1 (5.88)
Toxicology	1 (5.88)
Pharmacology & Pharmacy	1 (5.88)

Each country/region is a member of at least one of the Organization for Economic Co-operation and Development (OECD), G20 or EU memberships. Following the United Nations classification, the common denominator is that they are industrialized, developed, or developing countries. The USA is the undisputed leading country in the LM field in terms of both the number of published papers and the widespread impact of the publications.

Using the data in Table 4, Pearson correlation coefficient was calculated to determine correlations among *h*-index and some indicators. The following guideline was adopted to interpret the correlation magnitude: very strong >0.9, strong (0.7–0.9), moderate (0.5–0.7), low (0.3–0.5), and negligible (0–0.3), respectively [59]. The correlation between *h*-index and some indicators are given in Table 5 in which there is a strong correlation between the *h*-index and the number of papers published by the countries, a moderate correlation with the GDP *per capita*, and a low correlation with the average number of citations. Demir et al. [41] pointed out that there is a similar strong positive relationship between the publication productivity and GDP/GDP purchasing power parity (*r = 0.726 for GDP, r = 0.703 for GDP ppp)*.

**Table 4.** Top 25 most productive countries/regions of papers for the LM category during 2011–2020.

County/region	Paper		Average citation (*n*)	*h*-index		GDP *per capita* (US$) [60]
	*n*	Rank		Value	Rank	
USA	5 059	1	10.96	72	1	63.051
Germany	1 990	2	9.77	51	4	45.466
England	1 830	3	12.48	57	2	39.229
Australia	1 508	4	9.68	44	6	51.885
Italy	1 351	5	9.91	42	7	30.657
China	1 285	6	8.97	36	12	10.839
Switzerland	1 086	7	14.37	51	5	81.867
Japan	1 026	8	7.77	37	10	39.048
France	696	9	11.53	39	9	39.257
Netherlands	680	10	16.41	52	3	51.290
Canada	641	11	10.17	32	15	42.080
India	615	12	6.93	27	20	1.877
Spain	604	13	13.89	42	8	26.832
Brazil	484	14	10.05	30	18	6.450
Romania	398	15	2.09	13	25	12.813
South Korea	391	16	9.31	27	21	30.644
Turkey	380	17	6.19	22	24	7.715
Belgium	355	18	15.99	37	11	43.814
Scotland	353	19	11.99	29	19	37.460
Denmark	341	20	16.40	35	13	58.439
Portugal	313	21	13.19	31	16	21.608
Poland	304	22	13.43	31	17	15.304
New Zealand	298	23	10.46	27	22	38.675
Sweden	266	24	12.86	26	23	50.339
Austria	250	25	16.61	35	14	48.634

When the ranking in Table 4 is reconfigured by the *h*-index, it was observed to have changed relatively. The countries/regions with the most changing rankings are Austria (+11), Denmark (+7) and Belgium (+7), Romania (–10), India (–8), and Turkey (–7), respectively. Although India, Romania, and Turkey produced more papers than Austria, Denmark, and Belgium in terms of the number of papers, they stand behind these countries in terms of both their *h*-index and the average number of citations per paper. Even though Austria, Denmark, and Belgium had fewer articles than India, Turkey, and Romania, the widespread effects of their papers were greater. Meanwhile, some research showed that the level of cooperation in the published papers and the funding for the research had an impact on the visibility of papers [61–63]. The level of cooperation between the five leading countries/regions and the six other countries is given in Figure 3.

**Figure 4.**
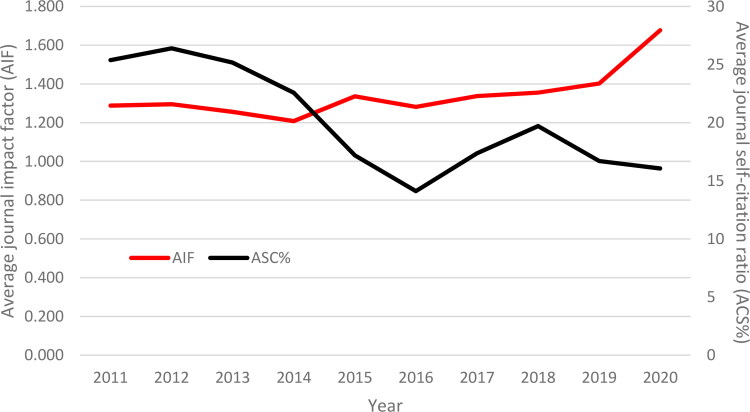
The variation of average journal impact factor (JIF) and self-citation ratio for the Legal Medicine (LM) category between 2011 and 2020. AIF: average journal impact; ACS%: average journal self-citation ratio.

Figure 3 indicates that the leading countries/regions work together significantly in terms of international cooperation, especially with the USA. On the other hand, the countries/regions with which the leading countries cooperated the most frequently are economically developed countries/regions. Austria, Belgium and Denmark are in a better position than the leading countries/regions in terms of international cooperation and they constantly cooperate with the leading and developed countries/regions. However, international cooperation for Romania, India, and Turkey are far behind Austria, Belgium, and Denmark. When the countries with which India cooperates were examined in the VOSviewer cluster structure, it was seen that India cooperates with countries such as USA, Saudi Arabia, Nepal and Malaysia. Turkey cooperates with the USA and the EU in principle, but the level of cooperation is limited, and Romania remains at a very limited level when compared with the other countries.

Table 6 shows the first three institutions funding the studies and the percentages of the papers funded in the five leading countries/regions and the six other countries. Forensic science research in the leading countries is supported by a variety of funds to a large extent. The EU member countries receive high support, especially from the EU commission. The USA, on the other hand, is heavily supported by research funds at the national level compared to the other leading countries. Research in Austria, Belgium, and Denmark is highly supported by EU funds. On the other hand, although Romania is a member of EU, it was not given sufficient support from these funds. Research in Turkey and India is supported at a low level and only by national funds.

**Table 6.** The percentage of papers funded for the top five countries/regions and the six other countries.

Country/region	Funding agencies	% of TP
USA	United States Department of Health Human Services	6.78
	National Institutes of Health USA	6.21
	National Institute of Justice office of Justice Programs	3.24
Germany	European Commission	3.17
	German Research Foundation	2.11
	Projekt DEAL	2.06
England	European Commission	5.36
	Research Innovation UK	5.14
	Engineering Physical Sciences Research Council	2.90
Australia	Australian Research Council	6.70
	Australian Government	3.52
	US National Institute of Justice	2.39
Italy	European Commission	3.78
	Swiss National Science Foundation	1.48
	Ministry of Education Universities and Research	1.33
Austria	European Commission	13.20
	Austrian Science Fund	10.80
	National Institute of Justice office of Justice Programs	3.20
Belgium	FWO	5.35
	European Commission	4.23
	Institute For The Promotion of Innovation	2.25
Denmark	European Commission	5.28
	Ellen and Aage Andersen Foundation	4.40
	Novo Nordisk Foundation	2.05
Romania	European Social Fund	1.51
	Romanian Government	1.51
	Consiliul National al Cercetarii Stiintifice	1.26
India	University Grants Commission India	5.85
	Department of Science Technology India	2.60
	Indian Council of Medical Research	1.79
Turkey	Tubitak Turkey	2.90
	Cukurova University	1.05
	Baskent University	0.79

TP: total paper.

**Table 5.** Pearson correlation matrix between *h*-index and some indicators.

Indicator	Pearson correction coefficient (*r*)	*P*-value
Paper number	0.798	0.000
GDP *per capita* (US$)	0.607	0.001
Average citations per paper	0.402	0.046

### Results and discussion for RQ3

Descriptive statistics for the JIFs in the category of LM between 2011 and 2020 are given in Table 7. As seen in Table 7, the average JIF in the LM category increased from 1.288 (median: 1.215) to 1.677 (median: 1.718) for 13 journals indexed regularly during the studied period.

**Table 7.** Descriptive statistics of the journal impact factor (JIF) for the 13 journals indexed regularly in the Legal Medicine (LM) category from 2011 to 2020.

JIF	2011	2012	2013	2014	2015	2016	2017	2018	2019	2020
Mean	1.288	1.295	1.256	1.208	1.336	1.282	1.337	1.355	1.402	1.677
Median	1.215	1.151	0.989	1.097	1.322	1.135	1.103	1.438	1.302	1.718
Maximum	2.587	2.686	2.597	2.714	2.862	2.382	2.815	2.996	2.652	3.271
Minimum	0.308	0.208	0.152	0.233	0.144	0.108	0.320	0.480	0.488	0.363
SD	0.765	0.821	0.740	0.789	0.820	0.731	0.775	0.768	0.694	0.846

The average JIF in the LM category increased by approximately 30.20% in the period of 2011–2020. This was expected because the JIF can vary depending on the field characteristics and time. This is called JIF inflation [54]. The main causes for this factor inflation are: the growth of the field, the growth in the average number of citations used per paper or the lower average citation age, the increase in the collaboration with other disciplines, the increased rate of citation for journals included in the WoS database, the increase in international collaborations and the increase in the number of authors per paper [54]. Table 1 indicates that all of the parameters that directly affect the JIFs, as pointed out by Althouse et al. [54], increased constantly in the LM category in the period of 2011–2020.

Meanwhile, it is generally desirable that JIFs should not excessively fluctuate from one year to another [64–68]. In a previous study, Smart [64] focussed on education journals, utilized Pearson’s correlation coefficients in order to estimate such fluctuations, and found that the JIF was stable. However, from a technical point of view, the use of Pearson correlation coefficients for quantifying such fluctuations may not be appropriate since yearly JIF datasets are longitudinal; hence, yearly observations are nested within the previous year’s observations [69]. As an alternative method of quantification, McGraw and Wong [69] suggested using the (ICC), which is a quantity that shows the level of agreement among observations. This quantity may be at values between 0 and 1: the highest values (close to 1) being preferred. Values less than 0.5 are indicative of poor reliability, values between 0.5 and 0.75 indicate moderate reliability, values between 0.75 and 0.9 indicate good reliability, and values greater than 0.9 indicate excellent reliability [70]. Journals in the LM category had an ICC estimate of 0.891 (95%CI: 0.793 to 0.958) for their impact factors over time, meaning that journals in this category had very stable impact factors.

The percentages of self-citations for each journal by the year are given in Table 2. There was no statistically significant correlation between the JIFs and the rates of self-citations. Figure 4 suggests that the rate of self-citations tended to decrease over the years. Larivière and Sugimoto [20] found that the percentage of self-citations across all disciplines remained at around 12% for the 2016 citation data for the papers published in 2014–2015 in the WoS database. However, they pointed out that the percentage varied widely by discipline, and they emphasized that these self-citation percentages may be higher in fields that have a limited number of journals, such as the level of specialized journals. Considering the average percentage of self-citations for the period of 2011–2020, *Rechtsmedizin* (50.98%), *Forensic Sci Int-Gen* (47.13%) and *Rom J Leg Med* (28.62%) stand out (Table 2).

**Figure 5.**
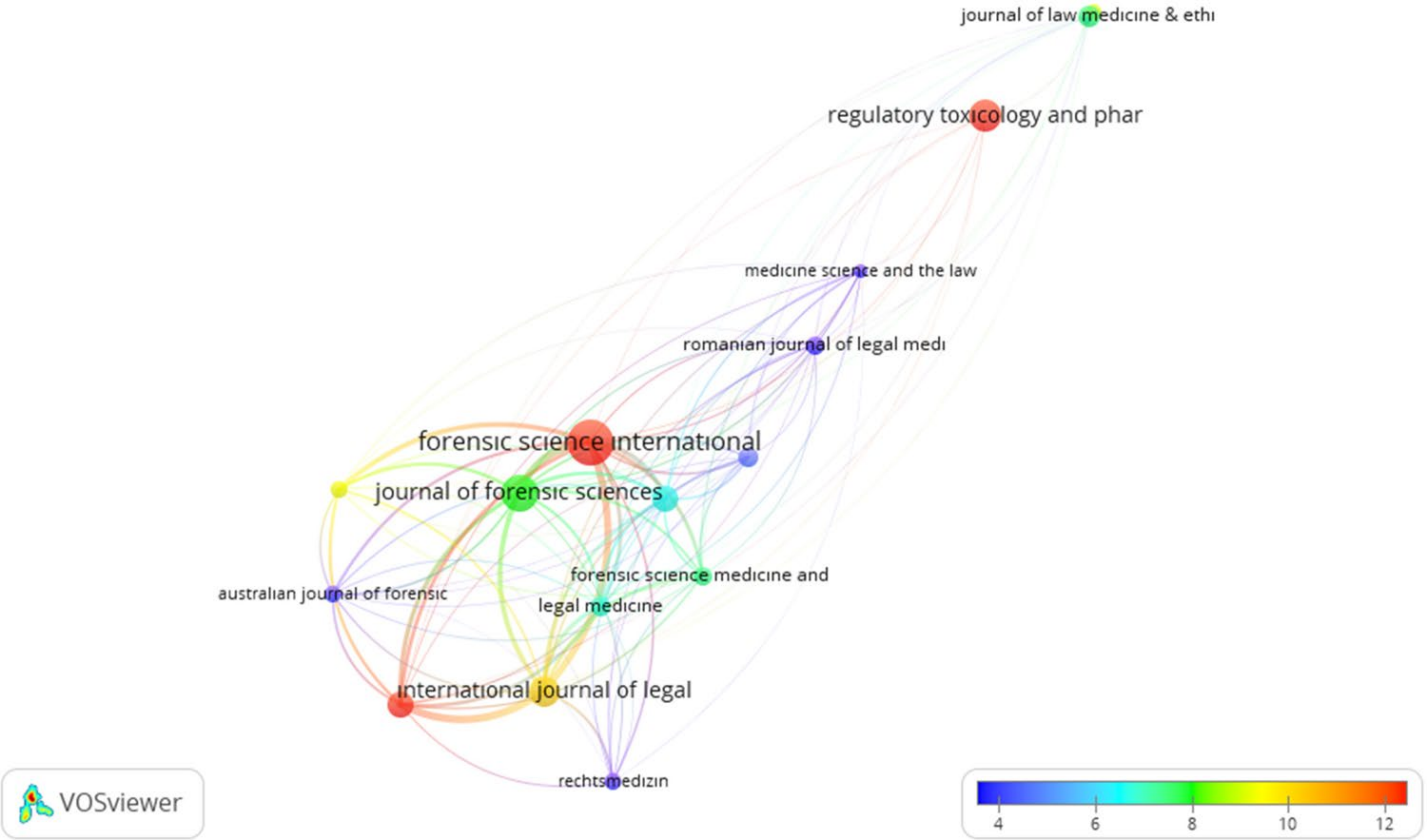
Overlay visualization for citation analysis of journals in the Legal Medicine (LM) category.

The relationship of networks created by considering the average citation number of the journals in the LM category is given in Figure 5. The number of average citations from blue to red (blue-green-yellow-red) increases, the circle size represents the number of papers published by the journals, and those with a high impact factor stand out as shown in Table 2. Figure 5 reveals an interesting correlation between the JIF and the number of papers in the journals from 2011 to 2020. It can be inferred that the journals with the higher impact factors (Q1 and Q2) publish more papers than journals with the lower impact factors (Q3 and Q4) in the LM category, which seems to be in agreement with the previous studies in the literature [71–74]. All in all, quality and quantity are positively correlated [75].

**Table 8.** Descriptive statistics of the journal impact factor (JIF) for the legal medicine (LM) category from 2011 to 2020 (for 17 regularly indexed journals).

Year	JIF		
	Mean ± SD	Median	Range
2011	1.288 ± 0.765	1.215	0.308–2.587
2012	1.295 ± 0.821	1.151	0.208–2.686
2013	1.256 ± 0.740	0.989	0.152–2.597
2014	1.208 ± 0.789	1.097	0.233–2.714
2015	1.336 ± 0.820	1.322	0.144–2.862
2016	1.282 ± 0.731	1.135	0.108–2.382
2017	1.337 ± 0.775	1.103	0.320–2.815
2018	1.355 ± 0.768	1.438	0.480–2.996
2019	1.402 ± 0.694	1.302	0.488–2.652
2020	1.677 ± 0.846	1.718	0.363–3.271

## Conclusions

The current paper focussed on the characteristics of the paper outputs for the LM category using the WoS database from 2011 to 2020, which is the world’s oldest, most widely used and authoritative database of research publications and citations. The distribution of the total number of the papers for the 25 most productive countries/regions have been discussed in terms of quality and quantity by some bibliometric indicators. Since academic journals are increasingly ranked and rated by their impact factors, the answers to the questions have been investigated, *“What is the trend and temporal stability of the JIFs?”* and “*What are the self-citation rates of the journals?”*.

In response to RQ1, the number of papers (the growth pattern represented by a linear model), the average number of pages of the papers (from 6.74 to 8.57), the average number of cited references in the papers (from 27.74 to 35.58), and the average number of authors per paper (from 4.19 to 5.15), the percentage of open access papers (from 4.99% to 23.12%) as well as international (from 16.78% to 20.44%) and domestic (from 35.52% to 41.48%) collaboration in the papers, tend to increase regularly. However, the productivity is limited when compared to the whole WoS database, since there has been no significant change in the number of the journals. This could be attributed to the small size of the field, fewer active researchers, and less pressure on the researchers to publish more, as Jones [6] stated. It is important to note that the LM field has a very close relationship with other disciplines. The first five fields in which the LM studies interact, and the percentage of interaction were Pathology (12.03%), Pharmacology & Pharmacy (9.89%), Toxicology (9.89%), Law (8.19%), and Genetics & Heredity (6.83%), respectively.

In response to RQ2, the distribution of the number of papers for the 25 most productive countries/regions was presented (see also Table 4) and extra data for these countries have been added, such as the average number of citations per paper, the *h*-index and the GDP *per capita* (US$) since the raw number of publications can be misleading when judging the merits of a country’s work. The USA is the undisputed leading country in terms of both the number of the published papers and the *h*-index. However, the results showed that the countries with the highest number of publications were not those that made the most impact in terms of the widespread impact of the publications (*h*-index). The research showed that the level of cooperation in the published papers and the funding for the research had a dramatic impact on the visibility of papers in the LM field. In this regard, Austria, Belgium, and Denmark were among the most successful countries while India, Romania and Turkey were far lower in both cases. These findings also indicated that small, well-governed nations with a long history of democracy were more effective at converting economic prosperity into high-quality science, as indicated by Allik et al. [76].

In response to RQ3, the average JIF increased by approximately 30.20% during the period of 2011–2020. This was an expected outcome because it can vary depending on the characteristics of the paper outputs and time. As it was clear from the response to RQ1, there was a regular increase in all of the variables affecting the JIF. It is also worth emphasizing that the impact factors have not fluctuated excessively over the years. Thus, the focus was on the temporal stability of the JIF values, and the findings indicated that the journals had an ICC estimate of 0.891 (95%CI: 0.793 to 0.958), meaning that they have been very stable. Another important issue was the percentage of self-citations for journals. There was no statistically significant relationship between the JIFs and the rates of self-citations. It should be noted that the rate of self-citations has decreased over the years. Meanwhile, the results revealed a correlation between the JIFs and the number of papers in the journals. It can be inferred that the journals with the higher impact factors (Q1 and Q2) published many more papers than those with the lower impact factors (Q3 and Q4) in the LM category, which seems to be in agreement with the previous studies [71–75].

Finally, the LM field working as the common denominator of medicine and law is a relatively small discipline when compared to the other medical fields. It goes without saying that every successful step taken in the academic sense will help the justice system as well as healthcare. However, it can also be inferred from the data obtained that the widespread effects of scientific productivity and publications are directly related to the geography and economic conditions of the countries. Consequently, underdeveloped and developing countries should make an effort to cooperate with the leading countries in this field and should allocate more research funds. Clearly, the fact that developed countries are more willing to cooperate with these countries will enable the LM field to take root on healthier ground.

## Limitations

This study had a few limitations. First, bibliometric indicators based on the number of citations are time-dependent indicators and can change over time. Second, only “articles and reviews” published in the LM category journals in the SCIE (these two document types representing 81.94% of total publications) were analyzed, since it was believed that the dataset predominantly represented the industry standard, even though other datasets are emerging. Important LM journals in other languages (such as Chinese and French) were not included. Finally, only bibliometric data from the WoS were selected; thus, some relevant publications might have been overlooked in this study. Based on the above limitations, further research might expand the scope of databases to include others, such as Scopus or PubMed.

## Data Availability

Data are available from the correspondence author upon reasonable request.
